# Association Between Risk Perception and Acceptance for a Booster Dose of COVID-19 Vaccine to Children Among Child Caregivers in China

**DOI:** 10.3389/fpubh.2022.834572

**Published:** 2022-03-16

**Authors:** Chenyuan Qin, Ruitong Wang, Liyuan Tao, Min Liu, Jue Liu

**Affiliations:** ^1^Department of Epidemiology and Biostatistics, School of Public Health, Peking University, Beijing, China; ^2^Research Center of Clinical Epidemiology, Peking University Third Hospital, Beijing, China; ^3^Institute for Global Health and Development, Peking University, Beijing, China; ^4^National Health Commission Key Laboratory of Reproductive Health, Peking University, Beijing, China

**Keywords:** COVID-19 vaccine, booster dose, children, risk perception, China

## Abstract

**Background:**

At present, the widespread variants and the weakened immunity provided by vaccines over time have further emphasized the importance of vaccination, boosters, and prevention efforts against COVID-19. Here, this study intends to investigate the acceptability of a booster dose of COVID-19 vaccine among child caregivers, aiming to explore the association between risk perception and child vaccine acceptance.

**Methods:**

This anonymous, national, cross-sectional survey was conducted for one week from November 12, 2021 in mainland China. The risk perception among child caregivers was assessed based on the Health Belief Model (HBM) and the individuals was equally divided into three levels according to the total preset scores of each perception dimension. Pearson χ^2^ test was used to compare the differences among participants stratified by sociodemographic characteristics, health status, knowledge factors and risk perception. Univariate and multivariate logistic regression models were performed to explore the associations between risk perception and the acceptance of a booster dose of COVID-19 vaccine.

**Results:**

A total of 88.46% of 1,724 participants were willing to accept the booster dose of the COVID-19 vaccine for their children. People who lived in central China (91.93%), had a high school or polytechnic school level education (93.98%), and had a history of COVID-19 vaccination (88.80%) were more likely to accept a booster dose of the COVID-19 vaccine for their children. The complicated vaccination process (24.5%) and uncertainty about the safety (16.5%) and efficacy (21.3%) of vaccines were the three main reasons for vaccine hesitancy among child caregivers. The acceptance of the booster dose of the COVID-19 vaccine was closely related to a higher level of perceived susceptibility (moderate: aOR = 1.56, 95% CI: 1.07–2.29, *P* = 0.022; high: aOR = 1.75, 95% CI: 1.06–2.89, *P* = 0.029) and high perceived benefit (high: aOR = 7.22, 95% CI: 2.63–19.79, *P* < 0.001). The results were stable in the sensitivity analysis.

**Conclusions:**

88.46% of child caregivers were willing to have a booster dose of COVID-19 vaccine to children, and the acceptance was closely associated with a higher level of perceived susceptibility and perceived benefit. The complicated vaccination process, uncertainty about the safety and effectiveness of COVID-19 vaccines were the main reasons for their hesitancy. Therefore, targeted public health measures to increase perceived susceptibility and benefit are still needed to meet the requirements of higher-level immunization coverage.

## Introduction

As a major public health concern, coronavirus disease 2019 (COVID-19) has raged around the world for nearly 2 years, with more than 260 million people struggling in this disease (1, 2). The invention of vaccines has proven to be a landmark in human history. Undoubtedly, vaccination is the most economical public health intervention to prevent and control infectious diseases, especially during the time without specific medicine (3). The recent emergence of the new variants (e.g., Delta variant and Omicron variant) and the weakened immunity provided by vaccines over time further emphasize the importance of vaccination, boosters, and prevention efforts against COVID-19 (4–7). According to the statistics of Our World in Data, approximately 326.22 million people in 21 countries have administered a booster dose of the COVID-19 vaccine as of December 9, 2021 (8).

Surveillance data from the WHO indicated that the proportion of confirmed cases in the 5 and 5–14 year age groups has increased from 1.0 and 2.5% in January 2020 to 2.9 and 19.9% in December 2021, respectively (9). Despite the observed milder illness and disease course of COVID-19 in children compared to adults, severe illness, death, and severe complications caused by COVID-19 among children are still worrying (10, 11). Additionally, Delta variate has led to a surge of infection in children, and the incidence rate of severe cases was 1%, and the case fatality rate was 1/10,000 (12). The increasing rate of children's severe illness and mortality has seriously threatened children's lives and could increase the risk of community transmission. The severe illness or death of children caused by COVID-19 will be even more damaging to families and societies (11). Therefore, the vaccination of children and adolescents is of great significance, and it is urgent to establish an immune barrier against this pandemic.

Children have very active and sensitive immune systems, which has led researchers to be more careful about the vaccination (13). Typically, every vaccine candidate, even for other conditions, would be evaluated first in adult patients and then in progressively younger ages (13). As a result, children received the initial COVID-19 vaccination much later than adults. Studies have shown the safety and effectiveness of child vaccination (14), most countries have now approved the initial COVID-19 vaccines for children aged 12 or older, and a few have already included some children under 12 (15–19). The United States already began vaccinating children and adolescents over the age of 5 in May 2021 (16). People aged 3–17 years were approved the emergency use of inactivated vaccines by Chinese government in July 2021 (17). Consent form for children and young people or parents and caregivers was also released by the UK government on September 21, 2021 (15). Previous studies have only assessed children's acceptance of the initial COVID-19 vaccine. Of 3,011 reproductive women, 8.44% were hesitant about the initial child vaccination against COVID-19 in China (20). In a cross-sectional survey consisting of 16 countries, only 69.2% of the respondents intended to accept the COVID-19 vaccines for their children. India, the Philippines, and Latin America generally have the highest vaccine acceptance to children, while the lowest acceptance was in Russia, the United States and Australia (21). Risk perception is people's subjective judgment of the characteristics and severity of a particular risk, which can ultimately influence people's behavior (22, 23). Children and adolescents under the age of 18, who make up more than a quarter of the global population, often rely on parental decision-making and guidance for their vaccination behavior (22).

At present, multiple variants have emerged globally, including five variants of concern that have been identified, and many countries have seen another outbreak with breakthrough cases reported from time to time (24). Due to the late start of childhood vaccination, few studies have linked risk perception of child caregivers with their willingness to give their children a booster dose of COVID-19 vaccines. The possible influencing factors of children's vaccination of a booster dose, especially parents' risk perceptions, are crucial for the subsequent formulation of policies to promote booster vaccination among children. Here, we intend to investigate the acceptability of a booster dose of COVID-19 vaccine among children's guardians, aiming to explore the adjusted association between risk perception and the acceptance of a booster dose of COVID-19 vaccines for children.

## Methods and Materials

### Study Design and Participants

This anonymous study was a national cross-sectional survey conducted for 1 week from November 12, 2021 in China. Our study relied on an online survey platform called Wen Juan Xing (25). As a professional data collection platform (Changsha Ranxing Information Technology Co., Ltd., Hunan, China), it contains more than 2.6 million fixed members (stratified by different regions, gender, age, occupation, etc.), and can provide authentic and reliable samples that meet the needs of scientific research (25). Our inclusion criteria were as follows: (1) Chinese citizens; (2) Having child aged under 18 years old; and (3) agreement to participate in this survey.

Since 87% of Chinese parents were willing to vaccinate their children with the initial COVID-19 vaccines (26), we calculated the sample size with α as 0.05 and confidence interval width as 0.087 (0.1P) by the exact (Clopper-Pearson) method using PASS 15 (NCSS LLC., Kaysville, UT, USA). The authors randomly allocated the questionnaires to be collected in 31 provinces according to the population proportion of 31 provinces in the *Statistical Yearbook 2021* (Supplementary File 1). A total of 1,724 valid questionnaires were collected, fully meeting the minimum sample size requirements.

### Assessment of Risk Perception

This research assessed the risk perception of COVID-19 among child caregivers using a survey tool based on the Health Belief Model (HBM), which was widely used to estimate vaccination intention in previous literature (27). The HBM is an important tool for people to carry out health behavior intervention programs, which can be used to study the factors affecting vaccination intention and promote the expected changes in vaccination behavior. This theoretical framework is constructed by five dimensions, including perceived susceptibility, perceived severity, perceived barriers, and perceived benefits, and cues to action (27). Here, the first four dimensions matched to a total of ten questions were we mainly used to determine the risk perception of the child caregivers. All questions were answered based on a three-point Likert scale (“very concerned or agree”, “concerned or not sure,” and “not concerned or disagree”), which were assigned scores of 3, 2, and 1, respectively. After summing up the individual's total preset score for each dimension, the individuals were equally divided into three levels.

### Acceptance for a Booster Dose COVID-19 Vaccine to Children Among Child Caregivers

All eligible participants answered the question “Are you willing to give your child a booster dose of COVID-19 vaccination if available?” The acceptance rate of a booster dose of COVID-19 vaccine to children was defined as the proportion of participants who answered “yes” of all child caregivers in this study. In this study, a child was defined as a minor under the age of 18.

### Covariates

In addition to the primary variates related to risk perception and attitudes toward a booster dose of COVID-19 vaccine for children, this research also included three groups of covariates that might influence the acceptability. Sociodemographic characteristics contained region, age group, sex, education, monthly household income per capita. history of chronic disease and history of COVID-19 vaccination were collected as information reflecting participants' health status. According to the previous studies (20), the knowledge of COVID-19 and COVID-19 vaccines of was child caregivers also investigated, including the sources of infection, common symptoms, prevention measures, susceptible population, vaccine safety and effectiveness, etc. For knowledge related questions, the correct answer was set in advance and represented 1 score, and the rest of the answers were given a score of 0. Finally, the score was divided into three classes by tertiles as “low”, “moderate” and “high”.

### Data Analysis

Frequencies and percentages were used to summarize the characteristics of categorical variables. The Pearson χ^2^ test was used to compare the differences among participants stratified by sociodemographic characteristics, health status, knowledge factors and risk perception. Univariate and multivariate logistic regression models were performed to explore the associations between risk perception and the acceptance of a booster dose of COVID-19 vaccine. To examine the robustness, the authors finally constructed four different models in sensitivity analyses. Model A is a univariate logistic regression model using crude odds ratios (cORs) to explain vaccine acceptance in different risk perception groups, and sociodemographic characteristics mentioned above were adjusted in model B. In model C, the authors controlled the remaining covariates based on model B—health status, knowledge factors and the other three aspects of risk perceptions. Furthermore, model D contained only the significant covariates in the Pearson χ^2^ test and the other three risk perceptions.

Based on model C, there were subgroup analyses that included different regions, age groups, sex, education, income, history of chronic disease, history of COVID-19 vaccination, knowledge score on COVID-19 and COVID-19 vaccination. *P* for interaction was calculated to test the possible interactions between risk perception related variates and covariates. All analyses in this study were performed by SPSS 26.0, and a *p*-value of >0.05 was indicated statistically significant.

## Results

### Characteristics of 1,724 Participants

Finally, 1,724 child caregivers were eligible for recruitment. Among them, 46.23% lived in eastern China, 50.29% were male, and 77.26% had at least a bachelor's degree. Participants under 40 years old accounted for a large proportion (Table 1). Of the 1,724 child caregivers, 57.66% had moderate perceived susceptibility, 51.33% and 78.31% had high perceived severity and high perceived severity, respectively. Regarding perceived barriers, nearly 80% of the participants perceived only a low degree of barriers to COVID-19 vaccination (Table 2).

**Table 1:** Acceptance for a booster dose of COVID-19 vaccine to children among 1,724 child caregivers in China by characteristics.

**Characteristics**	***N*** **(%)**	**Acceptance for a booster dose of COVID-19 vaccine to children (%)**	**χ^2^**	***P***
**Total**	1,724 (100)	1,525 (88.46)		
**Sociodemographic characteristics**				
**Region**			9.610	0.008^*^
Eastern	797 (46.23)	689 (86.45)		
Central	533 (30.92)	490 (91.93)		
Western	394 (22.85)	346 (87.82)		
**Age group (years)**			2.492	0.477
≤ 30	808 (46.87)	720 (89.11)		
31–40	748 (43.39)	655 (87.57)		
41–50	139 (8.06)	126 (90.65)		
>50	29 (1.68)	24 (82.76)		
**Sex**			0.019	0.889
Female	857 (49.71)	759 (88.56)		
Male	867 (50.29)	766 (88.35)		
**Education** ^**§**^			14.567	0.002^*^
High school or polytechnic school	83 (4.81)	78 (93.98)		
Junior college	215 (12.47)	178 (82.79)		
Bachelor's degree	1,332 (77.26)	1,192 (89.49)		
Postgraduate degree	94 (5.45)	77 (81.91)		
**Monthly household income per capita (RMB)**			0.902	0.924
≤ 3,000	29 (1.68)	26 (89.66)		
3,001–5,000	264 (15.31)	232 (87.88)		
5,001–10,000	836 (48.49)	735 (87.92)		
10,001–20,000	484 (28.07)	433 (89.46)		
>20,000	111 (6.44)	99 (89.19)		
**Health status**				
**History of chronic disease**			0.275	0.600
Yes	165 (9.57)	148 (89.70)		
No	1,559 (90.43)	1,377 (88.33)		
**History of COVID-19 vaccination**			7.532	0.006^*^
Yes	1,679 (97.39)	1,491 (88.80)		
No	45 (2.61)	34 (75.56)		
**Knowledge factors**				
**Knowledge score on COVID-19**			4.703	0.095
Low (score 0–4)	15 (0.87)	11 (73.33)		
Moderate (score 5–10)	784 (45.48)	687 (87.63)		
High (score 11–15)	925 (53.65)	827 (89.41)		
**Knowledge score on COVID-19 vaccination**			1.350	0.509
Low (score 0)	15 (0.87)	12 (80.00)		
Moderate (score 1–2)	937 (54.35)	826 (88.15)		
High (score 3)	772 (44.78)	687 (88.99)		
**Risk perception**				
**Perceived susceptibility**			8.583	0.014^*^
Low (score 2–3)	367 (21.29)	310 (84.47)		
Moderate (score 4–5)	994 (57.66)	884 (88.93)		
High (score 6)	363 (21.06)	331 (91.18)		
**Perceived severity**			8.351	0.015^*^
Low (score 2–3)	52 (3.02)	45 (86.54)		
Moderate (score 4–5)	787 (45.65)	678 (86.15)		
High (score 6)	885 (51.33)	802 (90.62)		
**Perceived barriers**			50.265	<0.001^*^
Low (score 3–4)	1,369 (79.41)	1,249 (91.23)		
Moderate (score 5–7)	325 (18.85)	253 (77.85)		
High (score 8–9)	30 (1.74)	23 (76.67)		
**Perceived benefit**			100.590	<0.001^*^
Low (score 3–4)	21 (1.22)	11 (52.83)		
Moderate (score 5–7)	353 (20.47)	268 (75.92)		
High (score 8–9)	1,350 (78.31)	1,246 (92.30)		

* *P < 0.05*.

§ *Those with education degrees below high school were categorized into high schools or polytechnic schools due to their limited quantity*.

**Table 2:** Risk perception among 1,724 child caregivers in China during COVID-19.

**Risk perception**	***N***	**%**
**Perceived susceptibility**		
Low (score 2–3)	367	21.29
Moderate (score 4–5)	994	57.66
High (score 6)	363	21.06
**Perceived severity**		
Low (score 2–3)	52	3.02
Moderate (score 4–5)	787	45.65
High (score 6)	885	51.33
**Perceived barriers**		
Low (score 3–4)	1,369	79.41
Moderate (score 5–7)	325	18.85
High (score 8–9)	30	1.74
**Perceived benefit**		
Low (score 3–4)	21	1.22
Moderate (score 5–7)	353	20.48
High (score 8–9)	1,350	78.31

**Figure 1:**
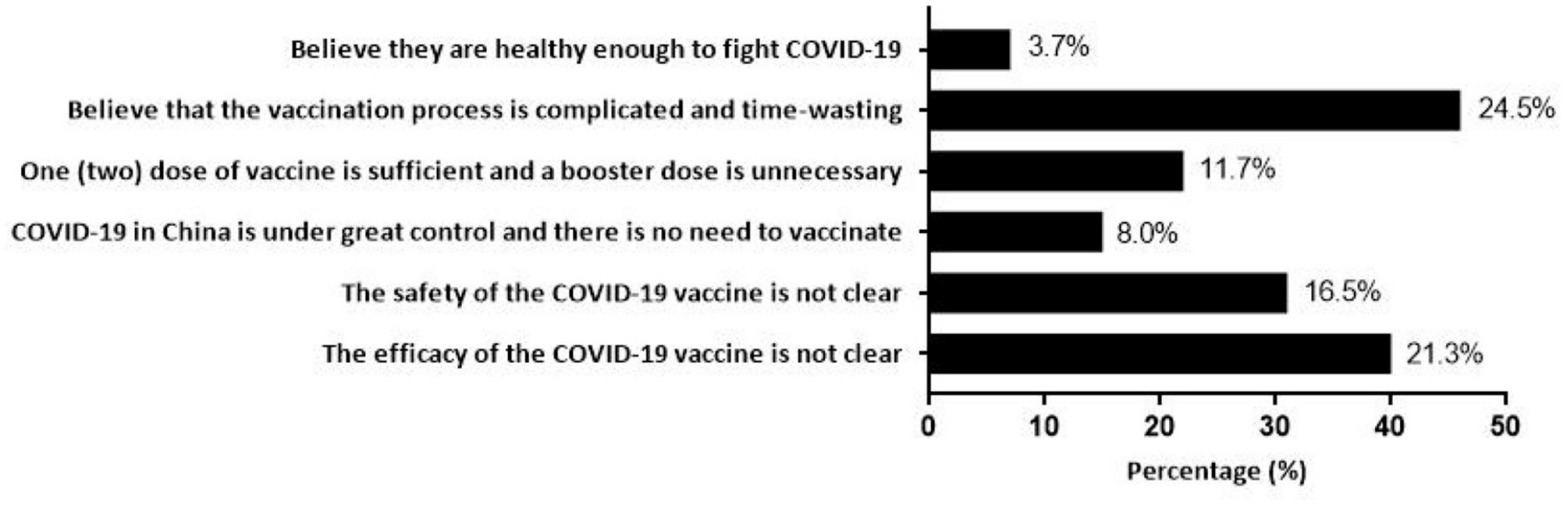
Reasons for hesitating to give a booster dose of COVID-19 vaccine to children among 188 child caregivers with COVID-19 vaccination history.

Of all participants, 88.46% (95% CI, 86.95–89.97%) were willing to accept the booster dose of COVID-19 vaccine for their children. The acceptance differences for a booster dose of COVID-19 vaccine to children were not statistically significant among different groups of age, sex, monthly household income per capita (RMB), history of chronic disease, and knowledge factors. People who lived in central China (91.93%), had a high school or polytechnic school level education (93.98%), and had a history of COVID-19 vaccination (88.80%) were more likely to accept a booster dose of the COVID-19 vaccine for their children (Table 1). Notably, there were 188 child caregivers who had a history of initial COVID-19 vaccination but did not want to give their children a booster dose. The complicated vaccination process and uncertainty about the safety and efficacy of vaccines were the main reasons for vaccine hesitancy (Figure 1). Furthermore, child caregivers with higher perceptions of susceptibility (91.18%), severity (90.62%) and benefit (92.30%) had a stronger willingness to receive a booster dose of vaccination for children. Additionally, vaccine acceptance was more likely to be observed in participants with low perceived barriers (91.23%) (Table 1).

### Association Between Risk Perception and Acceptance for a Booster Dose of COVID-19 Vaccine to Children

The association between risk perception and acceptance of a booster dose of COVID-19 vaccine to children is shown in Table 3. Without controlling for any confounding factor in model A, booster dose acceptance was associated with a higher level of perceived susceptibility (moderate: cOR = 1.48, 95% CI: 1.05–2.09; high: cOR = 1.90, 95% CI: 1.20–3.01), higher perceived benefit (moderate: cOR = 2.87, 95% CI: 1.18–6.98; high: cOR = 10.89, 95% CI: 4.52–26.24), and a low level of perceived barriers (low: cOR = 3.17, 95% CI: 1.33–7.54). The associations above remained stable in model B after controlling for sociodemographic characteristics, including region, age, sex, etc. Multivariate logistic regression of model C with all covariates adjusted displayed that the major factors related to the acceptance of the booster dose of COVID-19 vaccine were higher level of perceived susceptibility (moderate: aOR = 1.56, 95% CI: 1.07–2.29; high: aOR = 1.75, 95% CI: 1.06–2.89) and high perceived benefit (high: aOR = 7.22, 95% CI: 2.63–19.79). Model D showed similar association results when only covariates with statistical significance were included.

**Table 3:** The association between risk perception and the acceptance of a booster dose of COVID-19 vaccine to children among 1,724 child caregivers in China.

	**Model A**		**Model B**		**Model C**		**Model D**	
	**Crude odds ratio 95% CI)**	***P-*** **value**	**Adjusted odds ratio (95% CI)**	***P-*** **value**	**Adjusted odds ratio (95% CI)**	***P-*** **value**	**Adjusted odds ratio (95% CI)**	***P-*** **value**
**Perceived susceptibility**								
Low (score 2-3)	Reference		Reference		Reference		Reference	
Moderate (score 4-5)	1.48 (1.05-2.09)	0.027^*^	1.52 (1.06-2.17)	0.021^*^	1.56 (1.07-2.29)	0.022^*^	1.57 (1.08-2.28)	0.018^*^
High (score 6)	1.90 (1.20-3.01)	0.006^*^	1.83 (1.14-2.92)	0.012^*^	1.75 (1.06-2.89)	0.029^*^	1.84 (1.12-3.01)	0.016^*^
**Perceived severity**								
Low (score 2-3)	Reference		Reference		Reference		Reference	
Moderate (score 4-5)	0.97 (0.43-2.20)	0.937	1.04 (0.45-2.38)	0.933	0.67 (0.26-1.75)	0.414	0.59 (0.24-1.49)	0.265
High (score 6)	1.50 (0.66-3.44)	0.335	1.62 (0.70-3.76)	0.261	0.94 (0.36-2.46)	0.896	0.79 (0.31-2.00)	0.621
**Perceived barriers**								
Low (score 3–4)	3.17 (1.33–7.54)	0.009^*^	3.13 (1.29–7.59)	0.012^*^	1.57 (0.58–4.25)	0.373	1.44 (0.56–3.72)	0.452
Moderate (score 5–7)	1.07 (0.44–2.59)	0.882	1.02 (0.41–2.53)	0.963	0.59 (0.22–1.61)	0.305	0.59 (0.23–1.55)	0.289
High (score 8–9)	Reference		Reference		Reference		Reference	
**Perceived benefit**								
Low (score 3–4)	Reference		Reference		Reference		Reference	
Moderate (score 5–7)	2.87 (1.18–6.98)	0.020^*^	2.82 (1.11–7.16)	0.030^*^	2.10 (0.77–5.77)	0.149	2.15 (0.81–5.6)	0.124
High (score 8–9)	10.89 (4.52–26.24)	<0.001^*^	11.21 (4.46–28.13)	<0.001^*^	7.22 (2.63–19.79)	<0.001^*^	7.00 (2.64–18.56)	<0.001^*^

* *P < 0.05*.

*Model A is a univariate logistic regression model using crude odds ratios (cORs) to explain vaccine acceptance in different risk perception groups. Region, age group, sex, education, and monthly household income per capita were adjusted in model B. In model C, we adjusted the remaining covariates based on model B—history of chronic disease, history of COVID-19 vaccination, knowledge score on COVID-19, knowledge score on COVID-19 vaccination and the other three aspects of risk perceptions. Model D contained only the significant covariates in the Pearson χ2 test and the other three risk perceptions*.

**Figure 2:**
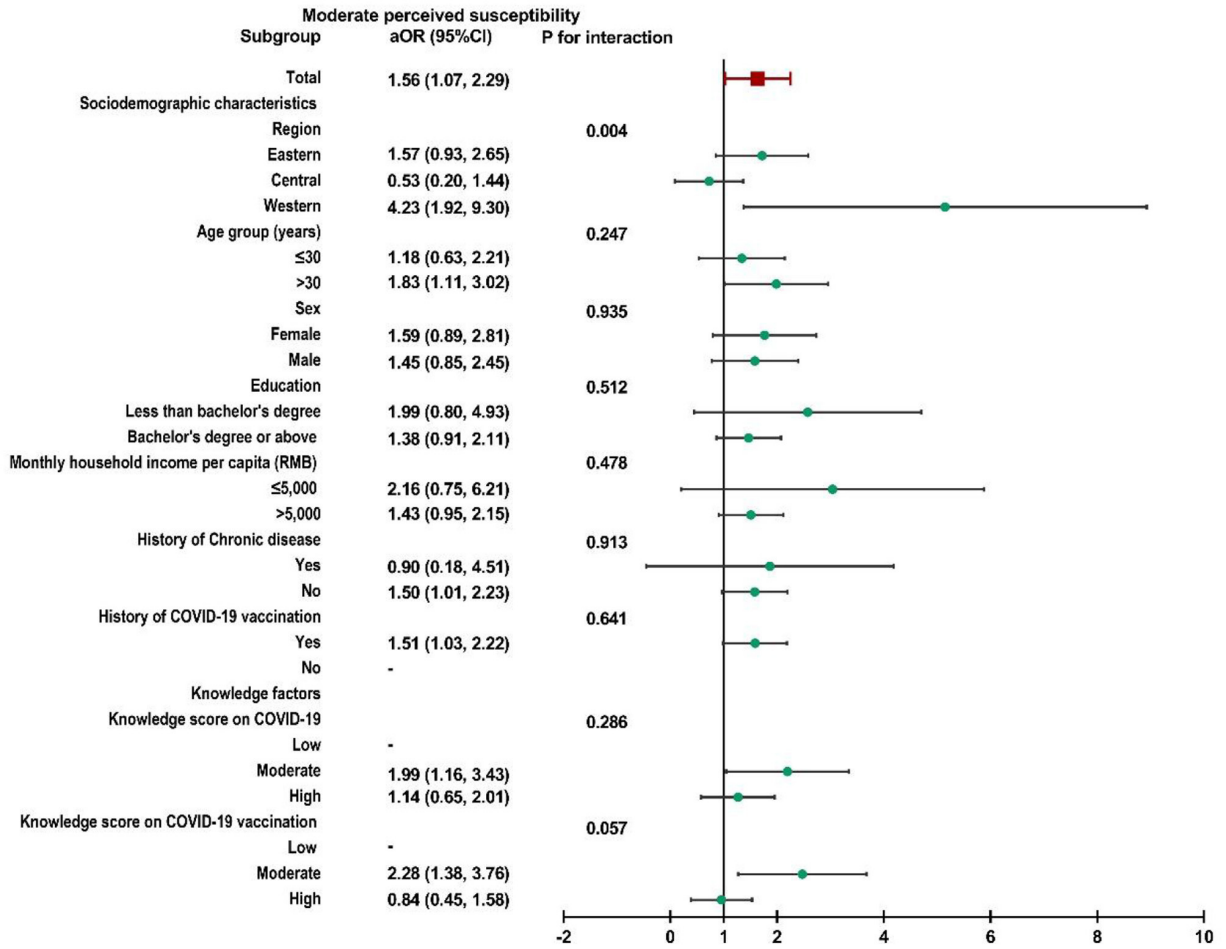
Subgroup analysis of the association between risk perception and acceptance of a booster dose of COVID-19 vaccine to children among caregivers with moderate perceived susceptibility.

**Figure 3:**
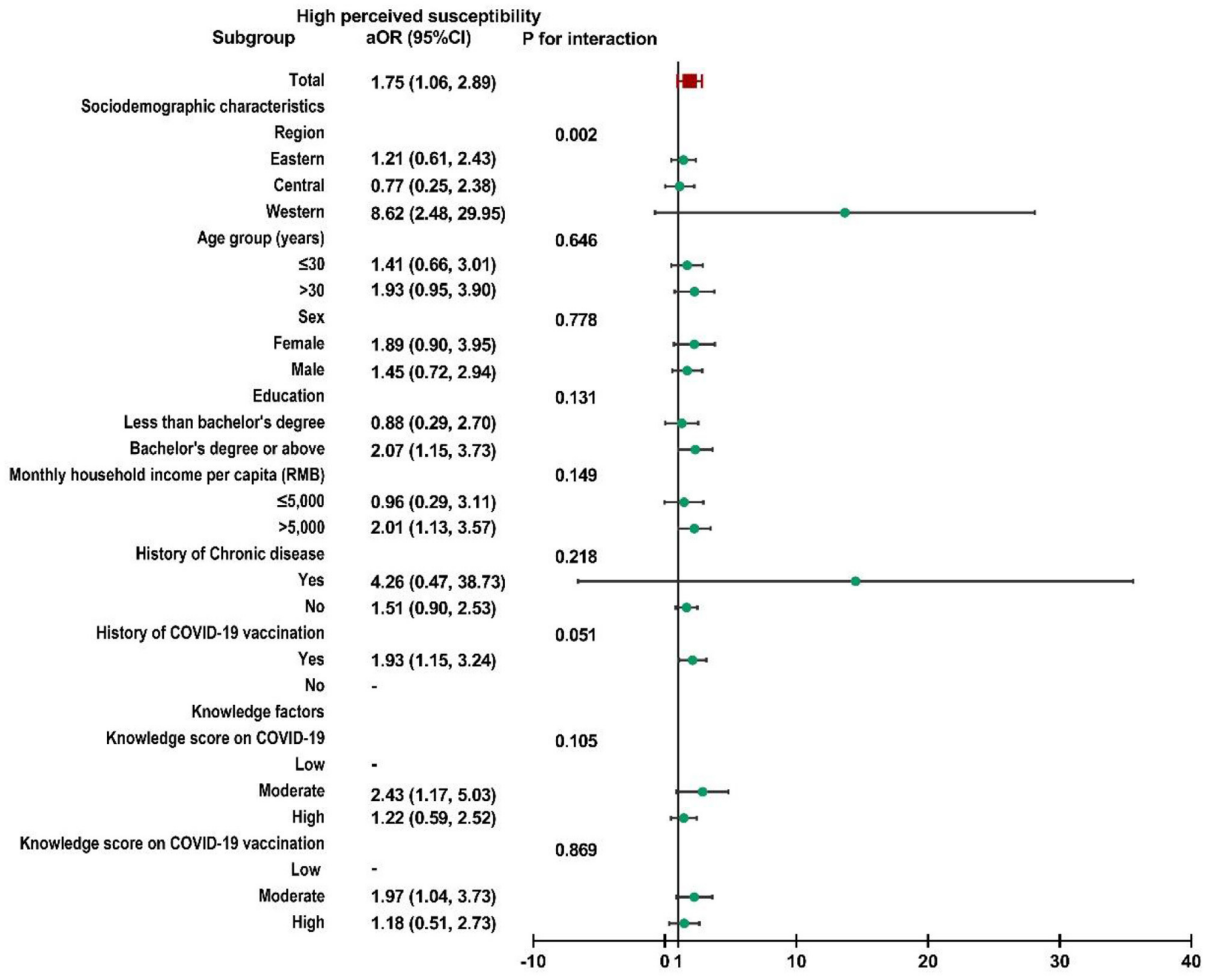
Subgroup analysis of the association between risk perception and acceptance of a booster dose of COVID-19 vaccine to children among caregivers with high perceived susceptibility.

Subgroup analysis was performed, and no interactions were found in most subgroups (Supplementary File 2). Child caregivers with moderate perceived susceptibility in western China were more likely to accept a booster dose of COVID-19 vaccine for their children than caregivers in the other two regions (*P* for interaction = 0.004, Figure 2). Similar results could also be observed in participants with high perceived susceptibility in western China (*P* for interaction = 0.002, Figure 3).

## Discussion

Currently, hundreds of countries around the world are still reeling from the COVID-19 pandemic. China, the United States, the United Kingdom, Japan, South Korea, Israel, and many other countries all have started to implement conditional vaccination policies for children (16–18, 28). Due to the particularity of children, initial COVID-19 vaccination is given much later than adults, and there are few studies on booster shots for children (13, 16–18, 28). Our research showed that 1,525 (88.46%) child caregivers were willing to give the booster dose of the COVID-19 vaccine for their children. But for the participants with COVID-19 vaccination history, complicated vaccination process and uncertainty about the safety and efficacy of vaccines were the main reasons they hesitated to vaccinate their kids. With all covariates adjusted, we found that the higher level of perceived susceptibility and perceived benefit, the more willing parents were to give booster shots to their children. To the best of our knowledge, this is the first nationwide study to explore the association between risk perception and acceptance of a booster dose of COVID-19 vaccine to children among child caregivers in China. A global survey demonstrated that the vaccine hesitancy changed over time because of the ever-changing public's risk perception of COVID-19 and information related to the vaccine safety and effectiveness (29). As a result, our findings have important theoretical implications for the subsequent development of strategies for the booster dose of COVID-19 vaccine in children.

According to our research, 88.46% of 1,724 participants were willing to accept the booster dose of the COVID-19 vaccine for their children. Due to its good safety and efficacy in adults (14), 55.3% of the world population has received at least one dose of a COVID-19 vaccine, and 8.28 billion doses have been administered globally as of December 9, 2021 (30). Evidence on the safety and effectiveness of COVID-19 vaccination for children and adolescents is gradually improving, and many countries and regions around the world have gradually implemented childhood vaccination programs (31–34). Multiple studies have reported mild to moderate adverse events in children vaccinated with COVID-19 vaccines, and vaccine-related severe adverse events were extremely rare (32–36). The results of a randomized, double-blind, controlled clinical trial for phase I/II in China suggest that inactivated COVID-19 vaccines BBIBP-CorV (32) and CoronaVac (37) could induce a strong humoral immune response against SARS-COV-2 in people under 18 years old. Ali et al. found that the mRNA-1273 vaccine had acceptable safety and was feasible and effective in preventing COVID-19 in adolescents aged 3–17 years, but it was currently difficult to assess the efficacy of the vaccine 14 days after completion of two doses of the vaccine accurately (33). In addition, two doses of the Pfizer/BioNTech vaccine were 93% (95% CI, 83–97%) effective in preventing COVID-19-related hospitalizations in children and adolescents aged 12–18 (31). However, studies on the safety and effectiveness of the booster dose of the COVID-19 vaccine in children and adolescents are lacking, and further time is needed to verify them.

Our results showed that the acceptance of a booster dose of vaccine in children was closely associated with a higher level of perceived susceptibility and perceived benefit, but not with perceived severity and perceived barriers. Parents' vaccination decisions are complex and multidimensional. Factors such as experience, emotion, risk perception and trust affect parents' attitudes and decision-making processes (38). Higher risk of infection, severe disease consequences, higher levels of perceived benefit, and lower perceived barriers were associated with higher vaccine acceptance among adults (39–42). And the concern about COVID-19 outbreaks and close attention to media coverage usually predicted a higher willingness to vaccinate (43). Min et al. found that women with lower perceived susceptibility (aOR = 2.44, 95%CI: 1.60–3.70 and lower perceived benefit (aOR = 4.59, 95%CI: 2.98–7.07) were more likely to be hesitant to vaccinate their kids. Association between parental acceptance of child vaccination and perceived severity was also not statistically significant (20). Another study showed that children with high perceived severity and susceptibility were much more likely to be vaccinated than those with low perceived threat (OR = 1.82, 95% CI: 1.21–2.72) (44). For young children, evidence suggested an association between vaccination and perceived disease susceptibility, but the evidence for an association between perceived disease severity and vaccination was weak (45). This may be because parents usually considered whether their child was susceptible to a disease before considering its severity (45). The association between perceived severity, perceived severity barriers and COVID-19 acceptance in children remains unclear. In conclusion, our findings suggested that increasing perceived risk to COVID-19 and the benefits of vaccination against COVID-19 at this stage is an effective way to increase children's willingness to be vaccinated against COVID-19.

Findings stratified by characteristics suggested that people who lived in central China, had a high school or polytechnic school level education, and had a history of COVID-19 vaccination were more likely to accept a booster dose of COVID-19 vaccine for their children. However, other studies found higher vaccine hesitancy for initial vaccination among child caregivers with lower education levels (46, 47). And the vaccination rates of initial COVID-19 vaccines have risen steadily with higher levels of education in the USA (48). 21.3% of 1,724 guardians of children in this research still have inadequate risk perception of vulnerability to COVID-19, which may be one of the reasons that prevents children from getting booster shots in the future. Targeted public health measures should be designated in accordance with local conditions to increase awareness of susceptibility to COVID-19 and the benefits to be gained from vaccination in children. While the global vaccination of children against COVID-19 has just begun, an early survey on children's willingness to receive booster shots can help identify potential barriers and remove barriers.

There are some limitations in our study. First, our risk perception is based on the HBM, so that the association between vaccination intention and risk perceptions among different theoretical models may not be comparable. Second, as our study was an online survey conducted in China, the results need to be interpreted with caution due to the limitations of the survey area and Internet users. Moreover, these findings may represent the attitudes of all children's guardians across the country toward the booster dose of COVID-19 vaccine, as only participants who already had kids were included. Also, this study didn't specifically investigate the age of each child.

## Conclusion

A total of 88.46% of 1,724 participants were willing to accept the booster dose of the COVID-19 vaccine for their children, and it was closely associated with a higher level of perceived susceptibility and perceived benefit. The complicated vaccination process, uncertainty about the safety and effectiveness of COVID-19 vaccines were the main reasons for their hesitancy. To promote vaccination, targeted public health measures should be designated to increase awareness of susceptibility to COVID-19 and the benefits to be gained from vaccination in children. Therefore, our findings have important theoretical implications for the subsequent development of strategies for the booster dose of COVID-19 vaccine in children.

## Data Availability Statement

The raw data supporting the conclusions of this article will be made available by the authors, without undue reservation.

## Ethics Statement

This study was approved by the Ethics Committee of Peking University Third Hospital. All participants signed informed consent forms and agreed to provide relevant data.

## Author Contributions

CQ, RW, and LT: conceptualization. CQ and JL: methodology and analysis. CQ: visualization and writing—original draft preparation. CQ, RW, ML, and LT: review and editing. JL: supervision. All authors have read and agreed to the published version of the manuscript.

## Funding

This study was funded by the National Natural Science Foundation of China (72122001; 71934002) and the National Science and Technology Key Projects on Prevention and Treatment of Major Infectious Disease of China (2020ZX10001002). The funders had no role in study design, data collection and analysis, decision to publish, or preparation of the paper. No payment was received by any of the coauthors for the preparation of this article.

## Conflict of Interest

The authors declare that the research was conducted in the absence of any commercial or financial relationships that could be construed as a potential conflict of interest.

## Publisher's Note

All claims expressed in this article are solely those of the authors and do not necessarily represent those of their affiliated organizations, or those of the publisher, the editors and the reviewers. Any product that may be evaluated in this article, or claim that may be made by its manufacturer, is not guaranteed or endorsed by the publisher.

## Supplementary Material

Supplementary File 1 A questionnaire about the acceptance of a booster dose of COVID-19 vaccine among child caregivers.

Supplementary File 2 Subgroup analysis of the association between risk perception and acceptance of a booster dose of COVID-19 vaccine to children for child caregivers.
