# Supplementary material for: Association Between Risk Perception and Acceptance for a Booster Dose of COVID-19 Vaccine to Children Among Child Caregivers in China
Source: Front Public Health. 2022 Mar 16;10:834572. doi: 10.3389/fpubh.2022.834572 (PMC8965812; doi:10.3389/fpubh.2022.834572)
Supplement: Supplementary File 1 — A questionnaire about the acceptance of a booster dose of COVID-19 vaccine among child caregivers. [file Table_1.DOCX]

**Supplemental file 1.**

**Questionnaire**

**Title: Association Between Risk Perception and Acceptance for a Booster Dose of COVID-19 Vaccine to Children Among Child Caregivers in China**

**Authors:** Chenyuan Qin^1^, Ruitong Wang^1^, Liyuan Tao^2^, Min Liu^1^, Jue Liu^1,3,4#^

**#Corresponding author:** Dr. Jue Liu, Department of Epidemiology and Biostatistics, School of Public Health, Institute for Global Health and Development, Peking University, Beijing 100191, China. Email: jueliu@bjmu.edu.cn, Tel: 86-10-82805146, Fax: 86-10-82805146

**A questionnaire about the acceptance of a booster dose of COVID-19 vaccine among child caregivers**

Thank you very much for participating in this survey. The main purpose of this questionnaire is to understand the willingness and influencing factors of a booster dose of COVID-19 vaccine in China. Please answer according to your true thoughts or actual situation.

This survey is only used for academic research and will be Not involved commercial use or privacy disclosure. This is an anonymous survey and no right or wrong answer. Please fill in the answer according to your actual situation. Thank you very much for taking up your precious time!

**Ⅰ. Sociodemographic characteristics and health status**

1. What is your gender? [Single choice] *

| ○ man |
| --- |
| ○ woman |

2. What is your level of education? [Single choice] *

| ○ Junior high school or below |
| --- |
| ○ High School or Technical Secondary School |
| ○ Junior college |
| ○ Bachelor’s degree |
| ○ Postgraduate degree |

3. Which age group do you belong to? [Single choice] *

| ○< 20 years old |
| --- |
| ○21-25 years old |
| ○26-30 years old |
| ○31-35 years old |
| ○36-40 years old |
| ○41-45 years old |
| ○46-50 years old |
| ○51-55 years old |
| ○56-60 years old |
| ○61-65 years old |
| ○66-70 years old |
| ○ > 70 years old |

4. Which average monthly income (RMB) group do you belong to? [Single choice] *

| ○ ≤3,000 |
| --- |
| ○ 3,001-5,000 |
| ○ 5,001-10,000 |
| ○10,001-20,000 |
| ○＞20,000 |

5. Do you have a child under the age of 18? [Single choice] *

| ○ Yes |
| --- |
| ○ No |

6. Do you have any chronic disease (such as cardiovascular disease, cancer, diabetes, chronic respiratory disease, etc.)? [Single choice] *

| ○ Yes |
| --- |
| ○ No |

7. Have you received the initial dose of COVID-19 vaccine? [single choice] *

| ○ Yes |
| --- |
| ○ No |

**II. Acceptance for a booster dose of COVID-19 vaccine to children**

8. Are you willing to give your child a booster dose of COVID-19 vaccination if available? [single choice] *

| ○ Yes |
| --- |
| ○ No or not sure |

9. Which of the following reasons are you not willing or sure to get a booster dose of COVID-19 vaccine for your kids? [Multiple optional] *

| □ The efficacy of the COVID-19 vaccine is not clear |
| --- |
| □ The safety of the COVID-19 vaccine is not clear |
| □ COVID-19 in China is under great control and there is no need to vaccinate |
| □ One (two) dose of vaccine is sufficient and a booster dose is unnecessary |
| □ Believe that the vaccination process is complicated and time-wasting |
| □ Believe they are healthy enough to fight COVID-19 |

**III. Knowledge of COVID-19 and vaccines**

10. Which of the following do you think is the source of infection for COVID-19? [Single choice] *

| ○ Patients |
| --- |
| ○ the asymptomatic |
| ○ Both |
| ○ Not clear |

11. Which of the following do you think are common symptoms of COVID-19? [Multiple optional] *

| □ Fever |
| --- |
| □ Diarrhea |
| □ Lack of power |
| □ Sore throat |
| □ Cough |
| □ Loss of sense of smell or taste |
| □ Rash |
| □ Chest pain |
| □ Not clear |

12. Which of the following do you think are ways to prevent COVID-19? [Multiple optional] *

| □ Cover your mouth and nose with tissue or towel when coughing or sneezing |
| --- |
| □ Wash hands frequently |
| □ Balanced diet |
| □ Get enough exercise and rest |
| □ Wearing a mask |
| □ Not clear |

13. Who do you think is at high risk for severe/critical COVID-19 [Multiple optional] *

| □ Older than 65 years |
| --- |
| □ With chronic disease |
| □ Heavy smoker |
| □ Third trimester and perinatal women |
| □ Immune deficiency |
| □ Obesity ( BMI≥30) |
| □ Not clear |

14. Do you think the following statement about COVID-19 is correct [Matrix Single choice] *

|  | Yes | Not sure | No |
| --- | --- | --- | --- |
| (1) People are generally susceptible to COVID-19. | ○ | ○ | ○ |
| (2) COVID-19 vaccination is free in China. | ○ | ○ | ○ |
| (3) At present, it is recommended that the booster dose vaccination should be complete can only be received 6 months after the whole vaccination. | ○ | ○ | ○ |
| (4) After vaccination, the protection decreases over time. | ○ | ○ | ○ |

**IV. Health beliefs on influenza COVID-19 and vaccination**

15. Do you have the following concerns? [Matrix Single choice] *

|  | Very Concerned | Concerned | Not Concerned |
| --- | --- | --- | --- |
| Are you concerned about getting COVID-19? | ○ | ○ | ○ |
| Are you concerned about family members getting COVID-19? | ○ | ○ | ○ |

16. Do you agree with this statement? [Matrix Single choice] *

|  | Agree | Not Sure | Disagree |
| --- | --- | --- | --- |
| (1) People who get COVID-19 are more likely to get severe illness. | ○ | ○ | ○ |
| (2) When you get COVID-19, your family's health may be at risk. | ○ | ○ | ○ |
| (3) A booster dose of COVID-19 vaccine can cause infection. | ○ | ○ | ○ |
| (4) It is not safe to get a booster dose against COVID-19. | ○ | ○ | ○ |
| (5) It is not effective to get a booster dose against COVID-19. | ○ | ○ | ○ |
| (6) It is good to strengthen your health with COVID-19 vaccination. | ○ | ○ | ○ |
| (7) It is good for family health when vaccinating a booster dose. | ○ | ○ | ○ |
| (8) A booster dose can provide better protection against COVID-19. | ○ | ○ | ○ |
