# Supplementary material for: Association Between Risk Perception and Acceptance for a Booster Dose of COVID-19 Vaccine to Children Among Child Caregivers in China
Source: Front Public Health. 2022 Mar 16;10:834572. doi: 10.3389/fpubh.2022.834572 (PMC8965812; doi:10.3389/fpubh.2022.834572)
Supplement: Supplementary File 2 — Subgroup analysis of the association between risk perception and acceptance of a booster dose of COVID-19 vaccine to children for child caregivers. [file Table_2.DOCX]

**Supplemental file 2** Subgroup analysis of the association between risk perception and acceptance of a booster dose of COVID-19 vaccine to children for child caregivers

caregivers

| **Subgroup** | **Moderate perceived susceptibility** | ***P* for** **interaction** | **High**  **perceived susceptibility** | ***P* for interaction** | **Moderate perceived severity** | ***P* for interaction** | **High perceived severity** | ***P* for interaction** | **Low**  **perceived barriers** | ***P* for interaction** | **Moderate perceived barriers** | ***P* for interaction** | **Moderate perceived benefit** | ***P* for interaction** | **High**  **perceived benefit** | ***P* for interaction** |
| --- | --- | --- | --- | --- | --- | --- | --- | --- | --- | --- | --- | --- | --- | --- | --- | --- |
| **Total** | 1.56 (1.07, 2.29) | | 1.75 (1.06, 2.89) | | 0.67 (0.26, 1.75) | | 0.94 (0.36, 2.46) | | 1.57 (0.58, 4.25) | | 0.59 (0.22, 1.61) | | 2.10 (0.77, 5.77) | | 7.22 (2.63, 19.79) | |
| **Sociodemographic characteristics** | | | |  |  |  |  |  |  |  |  |  |  |  |  |  |
| **Region** |  | **0.004*** |  | **0.002*** |  | 0.234 |  | 0.162 |  | 0.913 |  | 0.400 |  | 0.458 |  | 0.581 |
| Eastern | 1.57 (0.93, 2.65) | | 1.21 (0.61, 2.43) | | 0.65 (0.15, 2.76) | | 1.09 (0.25, 4.69) | | 1.31 (0.36, 4.73) | | 0.39 (0.11, 1.43) | | 3.12 (0.70, 13.97) | | 10.51 (2.37, 46.52) | |
| Central | 0.53 (0.20, 1.44) | | 0.77 (0.25, 2.38) | | 0.26 (0.02, 2.71) | | 0.24 (0.02, 2.63) | | - | | - | | 0.94 (0.15, 6.12) | | 3.80 (0.58, 24.81) | |
| Western | 4.23 (1.92, 9.30) | | 8.62 (2.48, 29.95) | | 2.33 (0.40, 13.72) | | 4.35 (0.72, 26.48) | | 3.10 (0.37, 26.22) | | 1.68 (0.19, 14.85) | | 2.40 (0.05, 113.61) | | 6.89 (0.15, 310.01) | |
| **Age group (years)** | | 0.247 |  | 0.646 |  | 0.154 |  | 0.146 |  | 0.247 |  | 0.161 |  | 0.306 |  | 0.823 |
| ≤30 | 1.18 (0.63, 2.21) | | 1.41 (0.66, 3.01) | | 1.76 (0.51, 6.07) | | 1.72 (0.50, 5.94) | | 2.56 (0.57, 11.44) | | 1.04 (0.23, 4.80) | | 3.04 (0.55, 16.82) | | 9.53 (1.72, 52.73) | |
| >30 | 1.83 (1.11, 3.02) | | 1.93 (0.95, 3.90) | | 0.24 (0.05, 1.23) | | 0.48 (0.10, 2.43) | | 0.94 (0.22, 3.97) | | 0.33 (0.08, 1.40) | | 2.30 (0.57, 9.22) | | 9.10 (2.25, 36.72) | |
| **Sex** |  | 0.935 |  | 0.778 |  | 0.225 |  | 0.352 |  | 0.233 |  | 0.151 |  | 0.084 |  | 0.054 |
| Female | 1.59 (0.89, 2.81) | | 1.89 (0.90, 3.95) | | 1.61 (0.51, 5.11) | | 1.80 (0.55, 5.85) | | 0.95 (0.18, 5.09) | | 0.33 (0.06, 1.75) | | 1.00 (0.26, 3.84) | | 2.96 (0.77, 11.37) | |
| Male | 1.45 (0.85, 2.45) | | 1.45 (0.72, 2.94) | | 0.24 (0.04, 1.42) | | 0.41 (0.07, 2.42) | | 2.38 (0.62, 9.20) | | 1.06 (0.27, 4.15) | | 12.05 (1.21, 119.72) | | 47.70 (4.81, 473.40) | |
| **Education** | | 0.512 |  | 0.131 |  | 0.999 |  | 0.998 |  | 0.802 |  | 0.929 |  | 0.189 |  | 0.230 |
| Less than bachelor’s degree | 1.99 (0.80, 4.93) | | 0.88 (0.29, 2.70) | | - | | - | | 1.12 (0.09, 14.16) | | 0.87 (0.07, 11.48) | | 0.71 (0.09, 5.80) | | 2.96 (0.37, 23.78) | |
| Bachelor’s degree or above | 1.38 (0.91, 2.11) | | 2.07 (1.15, 3.73) | | 0.89 (0.31, 2.50) | | 1.31 (0.46, 3.70) | | 1.59 (0.52, 4.85) | | 0.51 (0.17, 1.56) | | 2.53 (0.78, 8.18) | | 8.44 (2.61, 27.28) | |
| **Monthly household income per capita (RMB)** | | 0.478 |  | 0.149 |  | 0.830 |  | 0.424 |  | 0.311 |  | 0.363 |  | 0.570 |  | 0.820 |
| ≤5,000 | 2.16 (0.75, 6.21) | | 0.96 (0.29, 3.11) | | 0.39 (0.02, 8.07) | | 0.58 (0.03, 12.23) | | 4.78 (0.23, 99.65) | | 1.80 (0.09, 35.01) | | 2.39 (0.26, 21.86) | | 10.87 (1.17, 100.71) | |
| >5,000 | 1.43 (0.95, 2.15) | | 2.01 (1.13, 3.57) | | 0.73 (0.26, 2.03) | | 1.00 (0.36, 2.81) | | 1.32 (0.44, 3.98) | | 0.47 (0.15, 1.43) | | 2.56 (0.79, 8.30) | | 8.04 (2.49, 25.93) | |
| **Health status** | |  |  |  |  |  |  |  |  |  |  |  |  |  |  |  |
| **History of chronic disease** | | 0.913 |  | 0.218 |  | 0.968 |  | 0.231 |  | 0.999 |  | 0.999 |  | 0.999 |  | 0.999 |
| Yes | 0.90 (0.18, 4.51) | | 4.26 (0.47, 38.73) | | 0.50 (0.07, 3.83) | | 1.94 (0.19, 19.61) | | - | | - | | - | | - | |
| No | 1.50 (1.01, 2.23) | | 1.51 (0.90, 2.53) | | 0.56 (0.18, 1.78) | | 0.72 (0.22, 2.31) | | 2.25 (0.79, 6.44) | | 0.84 (0.29, 2.46) | | 2.70 (0.92, 7.92) | | 9.07 (3.10, 26.53) | |
| **History of COVID-19 vaccination** | | 0.641 |  | 0.051 |  | 0.759 |  | 0.644 |  | 0.444 |  | 0.389 |  | 0.999 |  | 0.999 |
| Yes | 1.51 (1.03, 2.22) | | 1.93 (1.15, 3.24) | | 0.67 (0.25, 1.79) | | 0.95 (0.35, 2.56) | | 1.31 (0.45, 3.87) | | 0.48 (0.16, 1.44) | | 1.90 (0.68, 5.32) | | 6.12 (2.19, 17.09) | |
| No | - | | - | | - | | - | | - | | - | | - | | - | |
| **Knowledge score on COVID-19** | | 0.286 |  | 0.105 |  | 0.166 |  | 0.052 |  | 0.637 |  | 0.836 |  | 0.258 |  | 0.177 |
| Low | - | | - | | - | | - | | - | | - | | - | | - | |
| Moderate | 1.99 (1.16, 3.43) | | 2.43 (1.17, 5.03) | | 1.11 (0.36, 3.45) | | 1.59 (0.49, 5.16) | | 1.92 (0.48, 7.73) | | 0.80 (0.20, 3.26) | | 1.38 (0.28, 6.74) | | 5.10 (1.04, 24.99) | |
| High | 1.14 (0.65, 2.01) | | 1.22 (0.59, 2.52) | | 0.19 (0.02, 1.59) | | 0.25 (0.03, 2.07) | | 1.86 (0.41, 8.36) | | 0.65 (0.14, 2.96) | | 6.49 (1.30, 32.45) | | 20.79 (4.21, 102.71) | |
| **Knowledge score on COVID-19 vaccination** | | 0.057 |  | 0.869 |  | 0.505 |  | 0.402 |  | 0.667 |  | 0.418 |  | 0.974 |  | 0.759 |
| Low | - | | - | | - | | - | | - | | - | | - | | - | |
| Moderate | 2.28 (1.38, 3.76) | | 1.97 (1.04, 3.73) | | 1.01 (0.34, 2.97) | | 1.23 (0.41, 3.68) | | 2.09 (0.60, 7.28) | | 0.82 (0.24, 2.82) | | 2.11 (0.59, 7.49) | | 5.50 (1.54, 19.66) | |
| High | 0.84 (0.45, 1.58) | | 1.18 (0.51, 2.73) | | 0.06 (0.00, 1.88) | | 0.10 (0.00, 2.96) | | 1.64 (0.30, 8.91) | | 0.56 (0.10, 3.17) | | 11.51 (0.76, 173.51) | | 54.76 (3.68, 815.54) | |

**P*<0.05, effect size was aOR and 95%CI
